# Optimized Distributed Proactive Caching Based on Movement Probability of Vehicles in Content-Centric Vehicular Networks †

**DOI:** 10.3390/s22093346

**Published:** 2022-04-27

**Authors:** Seungmin Oh, Sungjin Park, Yongje Shin, Euisin Lee

**Affiliations:** 1 Department of Computer Science and Engineering, Kongju National University, Cheonan 31080, Chungnam, Korea; smoh@kongju.ac.kr; 2 School of Information and Communication Engineering, Chungbuk National University, Cheongju 28644, Chungbuk, Korea; sjpark@chungbuk.ac.kr (S.P.); yjshin@chungbuk.ac.kr (Y.S.)

**Keywords:** content-centric networking, vehicular networks, pre-caching, mobility, roadside units (RSUs), content distribution

## Abstract

Content-centric vehicular networks (CCVNs) have considered distributed proactive caching as an attractive approach for the timely provision of emerging services. The naïve caching schemes cache all of the contents to only one selected roadside unit (RSU) for requested vehicles to decrease the data acquisition delay between the data source and the vehicles. Due to the high deployment cost for RSUs and their limited capacity of caching, the vehicular networks could support only a limited number of vehicles and a limited amount of content and thus decrease the cache hit ratio. This paper proposes a mobility-aware distributed proactive caching protocol (MDPC) in CCVNs. MDPC caches contents to the selected RSUs according to the movement of vehicles. To reduce the redundancy and the burden of caching for each RSU, MDPC distributes to cache partial contents by the movement pattern, the probability to predict the next locations (RSUs) on the Markov model based on the current RSU. For recovery of prediction failures, MDPC allows each RSU to request partial missing contents to relatively closer neighbor RSUs with a short delay. Next, we expand the protocol with traffic optimization called MDPC_TO to minimize the amount of traffic for achieving proactive caching in CCVNs. In proportion to the mobility probability of a vehicle toward each of the next RSUs, MDPC_TO controls the amount of pre-cached contents in each of the next RSUs. Then, MDPC_TO has constraints to provide enough content from other next RSUs through backhaul links to remove the delay due to prediction failures. Simulation results verify that MDPC_TO produces less traffic than MDPC.

## 1. Introduction

With the emerging demand for content-based applications, content-centric networking (CCN) has attracted attention for the next-generation communication paradigm [1]. In particular, based on mobile communication and intelligent vehicular technologies, content-centric vehicular networks (CCVNs) [2] are required to provide pleasant and safe driving as well as a variety of services such as multimedia entertainment and social interaction on the go [3,4]. Owing to the key services, e.g., on-the-road multimedia service, the scale of vehicular content increases significantly, on the other hand, the network will suffer from the amount of content and their requirement such as seamless services. In the future, the main type of content will be video content and its scale is larger and larger with user demand. It will cause a huge amount of traffic on the network. However, the network components have limited capacity (e.g., short communication range, intermittent connectivity, resources, etc.).

In the existing vehicular networks, each vehicle has its own on-board units (OBUs) so that it has a wireless networking function and can be provided content via the nearest roadside unit (RSU). The vehicles can connect with RSUs through vehicle-to-infrastructure (V2I) communication. The vehicles send their interests to obtain requested contents; then the RSUs could relay the requested contents provided from the remote content server(s). In the communication between the vehicles and the RSUs, the networks should support mobility as the vehicles are moving continuously and requesting a seamless content service. In other words, the requester vehicle should be provided contents from RSUs continuously. In spite of supporting mobility, the delay in receiving data from the RSU could increase when a vehicle sends a request for content because of a disconnection or moving to a new location before receiving the requested content. For the continuously moving vehicle, in-network caching has been considered an attractive approach for the CCVNs. It has two approaches to cache data in the networks: reactive and proactive approaches. In the reactive approach [5,6], if the requester vehicle disconnects from a current RSU, the RSU keeps caching content for the interest of the vehicle. When the vehicle reconnects to another new RSU, it informs the identity of the previous RSU to the new RSU for connection between the two RSUs. The new RSU requests the content being cached in the previous RSU. This approach also has the disadvantage of increased delay for the new RSU to start to transfer the contents to the vehicle.

Proactive caching [7,8,9] is a more efficient approach for content distribution. Proactive caching prefetches the contents of interest requested by vehicles ahead of time. The most basic way to cache contents is cache-all, which allows all caching units to cache all content passing through them. It is simple to implement; however, it makes high redundancy in the cache of RSUs. The redundancy causes additional delays in searching and processing. To efficiently cache contents, some studies [8,9] consider the mobility pattern of vehicles for prediction and select only several RSUs to cache contents. Unfortunately, they have still some problems. First, it also suffers from the cache limitation of each RSU; then it could support only a limited number of vehicles and a limited amount of contents. It can be a serious problem as the number of vehicles and the amount of content is gradually increased. Second, if the prediction based on the mobility pattern fails, it causes too significant delays in requesting and receiving the contents. If a vehicle connects an expected RSU to request successive contents, the RSU has no contents to provide and it should request or search the requested contents to the remote content provider (server).

This paper proposes a mobility-aware distributed proactive caching strategy in content-centric vehicular networks. To reduce the redundancy and the burden of caching for each RSUs, our previous work [10,11] distributes contents as much as the mobility pattern, which means the probability to predict the next locations (RSUs) of the Markov model based on the current location (RSU). For recovery of the prediction failure, the next RSUs are provided the information on the content distribution on them. With the information, each RSU could request partial missing contents to relatively closer RSUs with a short delay. However, the caching strategy of only using Markov model-based probability prediction has inefficient aspects in terms of the amount of content and partial missing contents transmitting delay. Additionally, network components have a limited capacity and limited bandwidth, so they can provide only limited content. Therefore, reducing backhaul traffic has become a new challenge. Thus, we expand the proposed proactive caching strategy through optimization, the mobility-aware distributed proactive caching protocol with traffic optimization called MDPC_TO to minimize the amount of traffic to achieve proactive caching in CCVNs. In proportion to the mobility probability of a vehicle toward each of the next RSUs, MDPC_TO controls the amount of pre-caching contents in each of the next RSUs. Then, MDPC_TO uses constraints to provide enough amount of content from other next RSUs through backhaul links to remove the delay due to prediction failures.

The rest of this paper is organized as follows. Section 2 introduces related works. In Section 3, the details of MDPC and MDPC_TO are presented. We evaluate our strategy and compare it with the existing schemes in Section 4. Finally, Section 5 concludes the paper.

## 2. Related Work

### 2.1. Vehicular Ad Hoc Networks (VANETs)

With the rapid development of mobile communication and intelligent transportation technologies, vehicular networks have emerged as a new paradigm [12,13]. Vehicular networks can provide not only pleasant and safe driving but also various kinds of services such as multimedia entertainment and social interactions on the go [3,4]. In existing vehicular networks, vehicles are equipped with on-board units (OBUs) so that they can communicate with each other by vehicle-to-vehicle (V2V) communications. Vehicles are also able to connect to roadside units (RSUs) by vehicle-to-infrastructure (V2I) communications. Nowadays, 10 percent of moving vehicles on the road are wirelessly connected. It is reported that 90 percent of vehicles would be connected by 2020 [14]. To satisfy the new demands, the design of next-generation vehicular networks becomes an important issue. However, there are new challenges to designing next-generation vehicular networks. First, with an ever-increasing scale of vehicular contents, network capacity becomes limited to efficiently deliver contents to vehicles. Second, because vehicles may pass through different locations for content delivery, how to manage content in different locations should be considered [15]. Third, due to the high velocity of vehicles, quality of experience (QoE) in vehicular networks should be improved to provide guaranteed services [2]. Recently, the VANETs have emerged into the promising paradigm of the Internet of Vehicles (IoVs) [16,17,18] according to the wave of Internet of Things (IoT).

### 2.2. Content Centric Networks (CCNs)

CCNs are communicated by request customers through the exchange of two kinds of packets: interest packets and data packets. When a consumer needs specific content, the consumer broadcasts an interest packet over the available network. A node holding required data responds to it with the data packet. The CCNs reduce packet collisions to prevent broadcast storm symptoms and provide a simple routine for retransmission when a packet is lost. The CCN network model also has three data structures: content store (CS), pending interest table (PIT), and forwarding information base (FIB). The CS is the space to store the content and the PIT is the table for interface tracking where the interest packets sent/received are delivered. The FIB is used for forwarding interest packets. The node that receives the interest packet checks if the requested content is in the CS list. If the content exists in the list, the data packet is transferred to the requester. Otherwise, the node searches the PIT for an interface that has transmitted an interest packet containing the same content name as the content name included in the interest packet and discards the interest packet. If not, a new PIT list including the interface on which the interest packet is received and the content name of the interest packet is included is created, and the interest packet is transferred to a node with data by using the FIB. When a node with matched data is found, the data packet is communicated to the requester through the PIT, and the node receiving the interest packet is utilized as a node for content distribution.

In the past few decades, the current Internet has evolved from an academic network into a global cyberinfrastructure. It is estimated that the annual network traffic of the Internet will surpass the zettabyte (i.e., 1024 exabytes) threshold in 2016, and video traffic would account for 79 percent of all consumer Internet traffic [19]. However, the current Internet architecture is centered on the host-to-host communication model, which makes it inefficient to support content distribution and information sharing. Accordingly, in recent years the networking community is actively designing novel Internet architectures from scratch, aiming at rectifying one or more of the current Internet’s drawbacks such as poor security and scalability. As a result, it is widely recognized that a content-centric network is a promising solution for meeting the needs of future networks [1,20].

Recently, most studies try to integrate vehicular networks and content-centric networks as CCNs [21,22] have been advocated to meet the challenges to design the next generation vehicular networks. In a content-centric vehicular network (CCVN), the content consists of two types of packets: interest packets and content packets. An interest packet consists of the naming information of the content. A vehicle can request content by sending an interest packet. The corresponding content packet will be sent to the vehicle if others have a replica of the content.

### 2.3. Caching in CCVNs

In content-centric vehicular networks, caching is to place storage in the data path to store data temporarily so that subsequent requests of the data network nodes simplify the data access mechanism by eliminating node addresses and facilitating caching at any network element such as routers and end-user devices. However, the main research challenges are defined by where and how to cache data. Ref. [23] describes the major challenges in finding the most appropriate network element to place data and identify the closest network node from the consumer holding the requested data. Refs. [20,24] investigate reactive and proactive (as called pre-caching) caching mechanisms and discuss the topic in terms of network topology, data popularity, and cooperation between network nodes.

Many studies have been conducted on proactive caching schemes. Ref. [25] proposed a method for information-centric networking (ICN) called selective neighbor caching that enhances seamless mobility in ICN, which selects an optimal subset of neighbor proxies that consider user mobility behavior. Similar to [8], Rao et al. [26] proposed a proactive caching approach for seamless user-side mobility support in NDN. The term proactive caching describes a class of strategies for taking action before a request is sent by a consumer. This concept aims at minimizing latency by pre-caching content nearby the consumer and maximizing performance by decreasing cache resource utilization. Such a strategy requires some degree of coordination and cooperation which can be separated into (i) path coordination—where data is cached at nodes nearby the delivery path such as WAVE [27] and (ii) neighborhood coordination—where data is cached at neighboring nodes and caching decisions are made locally (e.g., nearby the consumer or source node) such as a controller-based caching and forwarding scheme (CCFS) [28]. The proactive caching schemes are largely divided in to two categories: mobile pre-caching with AP and vehicular pre-caching with RSU [29,30].

#### 2.3.1. One Selection of One RSU

Work in [31,32,33] aims at reducing delay time at the RSU by selecting one RSU for caching. In [32], to reduce latency and contact time, pre-fetching is performed by determining that content that frequently occurs in interest is popular. Additionally, according to the caching policy, content that has no interest for a long time is removed. When the popularity of content does not exceed the popularity threshold by using interest request frequency, the number of hops, and the freshness of contents, the content is not cached. In [33], the authors deployed popular content in the RSU to minimize delay time. Ref. [33] cached content to the RSU according to the popularity of the contents. If the vehicle cannot download all the contents by the RSU, the remaining contents are downloaded through the BS. Additionally, a file distribution method for minimizing delay time is found through three algorithms: optimal, sub-optimal, and greedy. The work in [31] calculates the amount of content received through the position and speed of the vehicle, divides it into maximum payloads, and finds the number of chunks. This paper raise cache utilization and cache hit ratio through the calculation of the chunk number. The location where each chunk is requested is obtained through interest frequency, and the chunk predicted by each RSU is pre-cached to RSU. Delay and traffic are reduced because the request frequency for new content is calculated at a fixed value. However, they did not consider mobility because of highway scenarios [31,32,33].

#### 2.3.2. Selection of One RSU among Multiple RSUs

In [9,34,35], the authors use pre-fetching content to one RSU of a candidate of RSUs to reduce delay time and cache redundancy. Ref. [34] uses the LSTM (long short-term memory) algorithm to select the next RSU to reduce the network delay time by obtaining the content chunk information through the connection time, RSU coverage, and bandwidth. However, there is no recovery method when the vehicle does not move to the selected RSU using the LSTM algorithm. In [9], the mobility probability is used to reduce latency, server traffic, and cache redundancy. Mobility probability is determined using the Markov model and probability accuracy is determined using entropy. When the entropy value of the probability is low, it is considered that the accuracy of the probability is high, and pre-fetching is performed to the RSU. If the entropy value of the mobility probability subset is high, it goes to the upper level until the entropy value becomes low, and searches for the entropy subset that the entropy value is low. However, the delay was not considered in communication between the higher-level router and the lower-level router. They also did not propose a recovery method when they did not visit the most likely RSU. Ref. [35] increases the accuracy of mobility prediction, reduces backhaul traffic and latency, and improves cache hit ratio. Selected the content to predict the popularity of the content and pre-fetching it. Then, the bad/good of the trace information is calculated for each request period, and a 1st/2nd order Markov is selected to predict mobility. The selected Markov model is utilized to select the RSU to be pre-fetched. Refs. [9,34,35] reduces the duplication of caching but generates additional delay or backhaul traffic when not in the RSU that cached content.

#### 2.3.3. Selection of Multiple RSUs among Multiple RSUs

In [36,37,38], the content will be cached to several RSUs among several RSU candidates. Ref. [36] increased the rate of mobility prediction by maintaining the mobility information table to improve the cache hit ratio. The CRT updates its popularity so as to place more weight on the latest requests, and the NPT and RHT maintain/update the vehicle’s mobility to where and how long the vehicle has mobility. The CRT updated its popularity with more emphasis on the latest requests, and the NPT and RHT maintain/update where the vehicle will go and how long it will remain mobile. Therefore, the more popular content is pre-fetched in the RSU which is a destination of a vehicular and the cache hit ratio is increased. A mobility probability table associated with a Next AP and a timetable staying in each AP are utilized to consider where and how much pre-fetch is performed. They increase the cache hit ratio to reduce delay and traffic for improved QoE. Using a project called Mobility First to measure mobility, the predicted AP would pre-fetch the content. Refs. [37,38] improves download success probability by utilizing optimization. In [37], their strategy uses historical data of vehicular velocity, wireless bandwidth, and RSU location to determine the probability of successful delivery. The content is cached by using two algorithms. The content having the highest transmission success probability of the content is cached to an RSU and popularity and new content are considered. Ref. [38] uses bandwidth and storage at constraint to maximize the transfer success probability for each content. However, to increase the transmission probability of the content with the lowest transmission probability, some trade-offs are accepted. The content stored on the RSU was optimized to ensure a minimum success rate and eliminate the need for maximum storage. The contents request and mobility path selection were realized according to Zipf’s law. However, to combat the uncertainty of prediction, they pre-fetch redundantly at multiple RSUs.

## 3. The Proposed Protocol

### 3.1. Network Model and Protocol Overview

**Figure 1:**
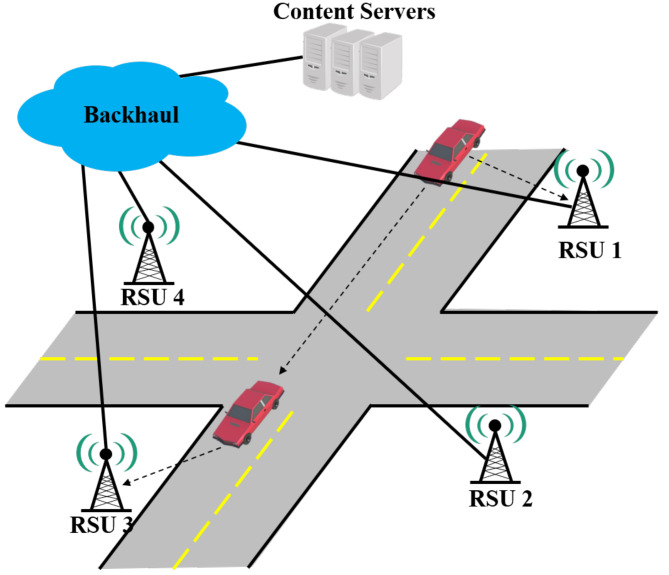
Overview of the proposed strategy on content-centric vehicular networks.

This paper considers the content-centric networking model [2] for the proposed strategy. Figure 1 shows CCN-based V2I communication using RSUs for content delivery to vehicles. In this model, when a vehicle enters the communication coverage of an RSU, the vehicle connects to the RSU infrastructure. When the vehicle needs a specific content, it transfers an interest packet for the content to the RSU. Vehicles communicate with RSUs using the IEEE 802.11p standard [39]. Each RSU is connected to a content server via a backhaul link and can receive content. In addition, RSUs are assumed to be connected and communicate with each other in a backhaul link. Additionally, each RSU can find an RSU with content via the FIB table [2]. When an RSU receives the content from the content server, the RSU transmits the content to the vehicle that has requested it.

Recently, as the size of content increases on the Internet, vehicles may not be able to receive the entire content with one RSU. The proposed strategy calculates the content size as much as possible that a vehicle can receive with one RSU, divides the obtained content by the chunk level, and caches some chunks of the content. In addition, content chunks are distributed and cached through transition probabilities derived by the Markov model [40]. The proposed strategy reduces the cache redundancy and backhaul traffic by distributed caching. When an RSU receives an interest packet from a vehicle, the RSU determines the transition probability matrix for the next RSUs by the Markov model. The RSU downloads the content chunk as much as possible that the vehicle can receive from content servers connected by the backhaul link. The current RSU, which has transferred content to the vehicle, calculates the transition probability matrix of the vehicle for distributed caching. The RSU calculates the content chunk number to be cached in the next RSUs in proportion to the transition probability and transmits the distributed information to the next RSUs. After receiving the distributed information, the next RSUs receive from the content server the content chunk of only the content chunk number included in the distributed information. The distributed information includes the identifiers of the next RSUs and the content chunk number to be cached in the RSUs. In short, a vehicle requests some content to a content server via a local RSU. The local RSU relays the request message and the movement probability to the server. According to the probability, the server distributes the requested content to the expected multiple next RSUs because the vehicle continuously moves to one of the next RSUs. Then, the vehicle reaches one of them; the reaching RSU transfers the cached content and re-requests the distributed content to the other RSUs.

### 3.2. Markov Model-Based Transition Probability

There are various ways to predict the next location where a vehicle will arrive soon. For example, vehicle navigation GPS, providing the path to a destination, can be used to predict the next location. However, the drivers might follow the preferred road rather than the pre-determined path information from the navigation system. Additionally, when driving on a familiar road, most drivers do not enter destination information in the navigation. Therefore, vehicle navigation could not guarantee the transition probability that a vehicle will arrive soon. The Markov model uses historical information about the vehicle to predict the next arrival location from the current location. Deriving transition probability based on the Markov model is a well-known method to predict mobility [40]. The Markov model predicts the future location based on the current location. With transition probability, this paper aims at caching more content to RSU with higher transition probability, less content to RSU with lower transition probability, and reducing caching redundancy and backhaul traffic. The RSU with which the vehicle communicated at the current location is $R_{i}$, and when the vehicle communicates with the RSU at the current location, the RSU with which the vehicle may next communicate at the current location is $R_{\left(i,j\right)}$. We define the number of $R_{\left(i,j\right)}$ as *n*. Therefore, $R_{j}^{i}$ can be presented as follows:

(1) $$R_{i}^{j}=\overset{n}{\underset{j=1}{⋃}}R_{\left(i,j\right)}=\left\{R_{\left(i,1\right)},R_{\left(i,2\right)},...,R_{\left(i,n\right)}\right\}.$$

Additionally, the probability of the vehicle moving from $R_{i}$ to $R_{\left(i,j\right)}$ is defined as $p_{ij}$. The number of times the vehicle has passed the communication range of the RSU at present is N${}_{i}$, and after passing the communication range of the RSU at present, the number of times the vehicle has passed the communication range of $R_{ij}$ is called $N(R_{i},R_{j})$. The movement probability $p_{ij}$ is defined as follows:

(2) $$p_{ij}=Pr\left(R_{i}|R_{\left(i,j\right)}\right)=\frac{N(R_{i},R_{\left(i,j\right)})}{N_{i}}.$$

Therefore, $p_{ij}$ is

(3) $$\sum_{j=0}^{n}p_{ij}=1.$$

### 3.3. The Amount of Caching Contents Based on the Probability

Currently, in order to cache contents for moving vehicles in the vehicular network, the existing studies consider either caching in the RSU with the highest transition probability or caching to all RSUs with the transition probability. However, such caching methods cause caching redundancy problems for the same contents in many RSUs as well as increasing backhaul traffic for content delivery to the many RSUs concurrently. In this paper, we partially cache content chunks based on transition probability to reduce the burden of caching in RSUs backhaul traffic. The existing studies focus only on who caches content and assume that the selected node predicts cache whole chunks of specific content. So, if the prediction fails, an additional cost is required for recovery. For instance, let us assume that there are RSUs $R_{A}$, $R_{B}$, $R_{C}$ as the next candidate RSUs of a vehicle. $R_{A}$ is selected by the current RSU and all chunks of the content are transferred to $R_{A}$. However, if the vehicle moves to the RSU $R_{B}$ or $R_{C}$, the caching content in $R_{A}$ is useless and the visiting RSU should request the contents to the content server. It requires additional delay and signaling messages. Moreover, the prediction requires the information gathered for a long time. If the historical location information (for vehicles) is insufficient, each next RSU has a similar value for transition probability.

### 3.4. Mobility Aware Distributed Proactive Caching (MDPC)

This subsection explains the details of our proposed strategy. We explain the process for MDPC and MDPC with entropy. MDPC calculates the amount of content that will be proactive to the RSU in that direction based on the transition probability. Based on the vehicle’s movement record, the probability of moving the vehicle was calculated using the Markov model. The Markov model is a cumulative recording method that records the number of times a vehicle has veered in a particular direction from that road relative to the number of times it has entered that road. Thus, the longer the learning time, the more accurate the probability can be.

#### 3.4.1. Caching Strategy

**Figure 2:**
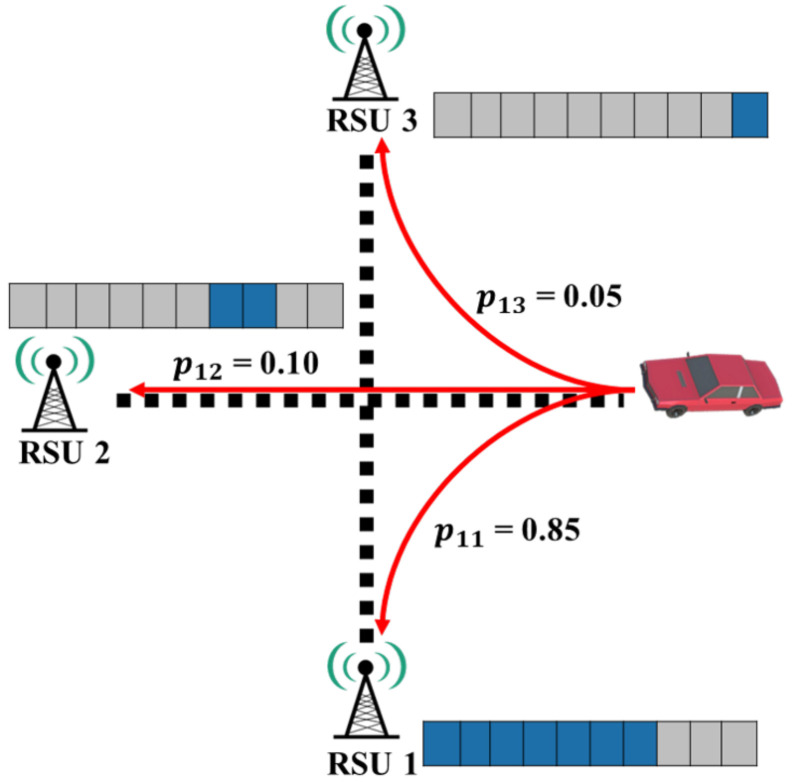
Transition probability example for each roadside unit.

This subsection shows the proposed chunk-level caching strategy based on the transition probability matrix as shown in Figure 2. Table 1 describes the content caching parameters. A roadside unit that has received an *interest* packet calculates the content-chunk size that the vehicle can receive within the RSU’s communication range through the vehicle’s speed and communication range. We can calculate the maximum number of chunks $C_{pre}$ as follows:

(4) $$C_{pre}=\frac{R_{RSU}}{V_{vehicle}}\times B_{V2I}.$$

where $R_{RSU}$ is the communication range of the roadside units, $V_{vehicle}$ is the velocity of a vehicle and is assumed to be constant, and $B_{V2I}$ means the bandwidth of the V2I communication. After the roadside unit $R_{i}$ calculates $C_{i}$, the unit downloads the content chunk from 1 to $C_{i}$ at the content server linked by the backhaul network. The RSU sends content chunks from 1 to $C_{i}$ to the vehicle and calculates the vehicle transition probability through Equation (2). The content chunk size $C_{pre(i,j)}$ cached by $R_{\left(i,j\right)}$ is calculated by the following:

(5) $$C_{pre(i,j)}=C_{pre}\times p_{ij},$$

where, $C_{pre}$ is $\sum_{j=1}^{J}C_{pre(i,j)}$.

$R_{i}$ determines the scope of content chunk for each next RSU according to the transition probability. To determine the scope, $R_{i}$ first calculates the maximum number of chunks as many as possible that it could relay with Equation (4). In addition, we use distributed points and $C_{r(i,j)}$ to calculate the content size cached to the next $R_{\left(i,j\right)}$ of the requested $R_{\left(i,j\right)}$. $C_{r(i,j)}$ is the content size to be transferred from the adjacent RSU, and $C_{r(i,j)}$ is calculated by the following:

(6) $$C_{r(i,j)}=\frac{B_{V2I}\times B_{Backhaul}}{B_{V2I}+B_{Backhaul}}+\left(\frac{C_{pre}}{B_{Backhaul}}-\frac{C_{pre(i,j)}}{B_{V2I}}\right)$$

The distributed point is calculated as follows:

(7) $$C_{i+1}=C_{pre(i,i+1)}+C_{r(i,i+1)}+C_{i},\left(j=i+1\right).$$

**Figure 3:**
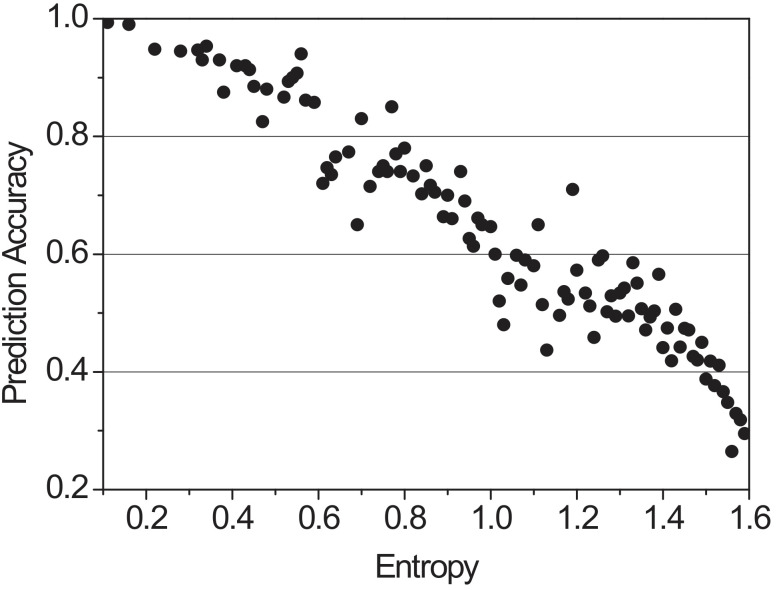
Correlation between entropy and prediction accuracy.

Therefore, the next $R_{\left(i,j\right)}$ caches the contents from $C_{i+1}$. The proposed strategy assigns the chunk number sequentially for simple number management. For the assignment of the numbers, we should consider the caching chunks not to be relayed by the previous RSU and the chunks to be cached by the new RSUs. $R_{i}$ assigns a content chunk number to be cached in $R_{\left(i,j\right)}$ sequentially from $R_{\left(i,j\right)}$ having the highest transition probability. $R_{i}$ sends a message containing the RSU ID of $R_{\left(i,j\right)}$ and the content chunk number of $R_{\left(i,j\right)}$. After $R_{\left(i,j\right)}$ receives the message to $R_{i}$, it searches for the content chunk number that matches its RSU ID and downloads the content chunk at the content server. Since the message for which $R_{\left(i,j\right)}$ is received contains information for the next $R_{\left(i,j\right)}$, $R_{\left(i,j\right)}$ can know the content chunk number to be cached to the neighbor $R_{\left(i,j\right)}$. For example in Figure 3 and Table 2, the values of $p_{ij}$ are 0.85, 0.10, and 0.05, respectively. We assume that the vehicle speed is 60 km/h, the communication range is 100 m, the V2I communication bandwidth is 20 Mbps, the content size is 1000 Mb, and the content chunk size is 1 Mb. By the probability $p_{ij}$, we assign 204, 24, and 12 chunks to $R_{\left(1,1\right)}$, $R_{\left(1,2\right)}$, and $R_{\left(1,3\right)}$. If the current RSU complete to relay the chunks from #1 to #240, the next RSUs continue to relay the maximum 240 chunks from #241. With the descending order of probabilities, $R_{\left(1,1\right)}$, $R_{\left(1,2\right)}$, and $R_{\left(1,3\right)}$ are assigned the chunks #241∼#444, #445∼#468, and #469∼#480, respectively.

#### 3.4.2. MDPC with Entropy Value (MDPC_E)

Entropy is a measure of uncertainty introduced by Claude Shannon in his paper [41]. With a discrete probability distribution $p=\{p_{1},p_{2},...,p_{n}\}$ where $p_{i}$≥0 and $\sum_{i=1}^{n}p_{i}=1$, the entropy *H* is calculated as

(8) $$H=-\sum p_{i}log_{2}p_{i}.$$

When the entropy value exceeds 0.5, it is estimated to be an inaccurate prediction. To support our use of entropy value as an inaccuracy, we simulated the correlation between prediction accuracy and entropy. The accuracy of the probabilities was calculated by setting success as if the vehicle had moved to the highest probability and fail if not. Figure 3 shows a graph of the accuracy measured according to the direction of the transition probability of the vehicle when the entropy value varies from 0.1 to 1.6. As a result, higher entropy means a lower prediction accuracy. In addition, When the prediction accuracy is greater than 90%, the entropy does not exceed 0.5.

With the given example *p* = {0.85, 0.10, 0.05}, the entropy *H* is −0.85 × log${}_{2}$0.85 − 0.10 × log${}_{2}$0.10 − 0.05 × log${}_{2}$0.05 ≈ 0.748. If the probability value is incorrect, caching is performed with a weight value for the most visited RSUs. As shown in Table 3, when the entropy value of $p_{ij}$ exceeds 0.5, $R_{i}$ adds the smallest probability of $p_{ij}$ to $p_{ij}$ of the most visited RSU when calculating $C_{ij}$. After that, $C_{i}$ determines the content chunk number of $R_{ij}$. The transition probability in Figure 3 will exceed 0.5 when the entropy value is calculated. Since $R_{11}$ is the most visited RSU, weighting is recalculated to perform more accurate caching. After recalculation, the entropy becomes −0.9 × log${}_{2}$0.9 − 0.1 × log${}_{2}$0.1 ≈ 0.469 < 0.5.

#### 3.4.3. Content Delivery

This subsection describes the procedure for content delivery between RSUs and a vehicle. The vehicle $V_{r}$, connecting a RSU $R_{i}$, moves to one RSU ($R_{\left(i,j\right)}$) among the set $R_{i}^{j}$. After the next RSU $R_{\left(i,j\right)}$ detects the movement of the vehicle $V_{r}$, the RSU transfers the content chunks pre-cached in it. As the RSU has the information on content distribution, the RSU could request the content not to cache to the other RSUs in the set $R_{i}^{j}$. After the RSU relays the content chunks received from the other RSUs to the vehicle, the RSU checks whether the last number $C_{ij}$ of chunks transferred is the same as the last chunk number of the requested content $C_{T}$. If they are the same, the RSU terminates the communication for the content. Otherwise, the RSU $R_{\left(i,j\right)}$ selects its next RSUs and calculates the transition probability and the chunk number assignment as described above.

**Figure 4:**
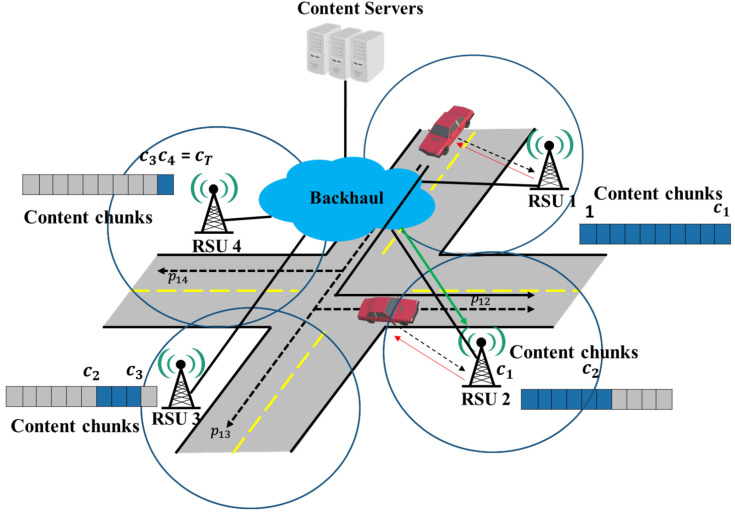
The proposed caching strategy for a vehicle and each RSU.

In our strategy as shown in Figure 4, as the first chunk is cached in the highest probability RSU, we consider the following two cases: the vehicle moves either to the highest RSU or to one of the other RSUs. In the case of the highest RSU, the RSU transfers the content chunks sequentially. However, if the vehicle moves to one of the other RSUs, which has a lower probability, it causes the chunk to reassemble the problem. Namely, it causes some chunks to be omitted in the chunk sequence. To recover the omitted chunks, the RSU assigns the chunk numbers for the next RSUs, including the omitted numbers.

### 3.5. MDPC with Optimization (MDPC_TO)

MDPC with optimization is a measure to minimize backhaul traffic by optimization using the transition probability in the intersection. Based on the transition probability of a vehicle, the MDPC_TO optimized the amount of pre-caching in a way that minimizes backhaul traffic. The RSU, where the vehicle enters next, brings content from other pre-cached RSUs while delivering optimized pre-cached content to the vehicle, providing the vehicle with a maximum amount of content.

#### 3.5.1. Objective Function

Let $R_{\left(i,j\right)}$ be one of the next RSUs when a vehicle *v* is staying in $R_{i}$. Thus, using $p_{i,j}$, the total backhaul traffic $U_{v}$ consumed by the vehicle as it moves in each direction can be calculated through Equation (9). The following function minimizes the total backhaul traffic under some constraints.

(9) $$min\;U_{v}=\sum_{j=1}^{J}\left(p_{ij}\times \left(\sum_{k=1}^{J}2d_{ik}-d_{ij}\right)\right)$$

(10) $$\begin{array}{ccc} s.t.-\frac{1}{r}d_{ij}+\sum_{k=1,k\neq j}^{J}\frac{1}{w}d_{ik} & \leq & 0,\forall j\in J \end{array}$$

(11) $$\begin{array}{ccc} d_{i(k+1)}-d_{ik} & \leq & 0,k>1 \end{array}$$

(12) $$\begin{array}{ccc} \sum_{j=1}^{J}d_{ij} & \leq & MIN(t_{dwell}\times w,MIN\left(\forall CAPA_{j}\right)) \end{array}$$

(13) $$\begin{array}{ccc} \forall d_{ij} & \geq & 0 \end{array}$$

#### 3.5.2. Constraints

The first constraint in Equation (10) means the limitation that the current RSU $R_{\left(i,j\right)}$, which communicates with the vehicle, requires obtaining pre-cached content from another $R_{\left(i,j\right)}$ wired to backhaul while the vehicle is provided with quantity $C_{pre(i,j)}$ of pre-cached content. This is because $R_{\left(i,j\right)}$ imports the remaining content before it could provide the vehicle with the quantity $C_{pre(i,j)}$ of pre-cached content before it can provide the remaining content immediately after providing $C_{pre(i,j)}$. If the constraint does not exist, the vehicle might receive pre-cached content from $R_{\left(i,j\right)}$ and receive nothing while importing the remaining content from $R_{\left(i,j\right)}$ other than $R_{\left(i,j\right)}$, which in turn leads to a delay.

Equation (11) describes that the $R_{\left(i,j\right)}$ with a higher transition probability must pre-cache the greater amount based on $p_{ij}$ arrayed with the large of the transition probability. If a large quantity of pre-caching is performed on an $R_{\left(i,j\right)}$ that is less likely to be moved, it will be more frequent for other $R_{\left(i,j\right)}$ to take content from $R_{\left(i,j\right)}$ because the vehicle is less likely to go to other $R_{\left(i,j\right)}$. Therefore, we had a constraint because of the increase in backhaul traffic. This can result in a small amount of pre-cached content being very small for $R_{\left(i,j\right)}$ that is sufficient. Backhaul traffic may increase to import the remaining content from other $R_{\left(i,j\right)}$ because the vehicle is likely to enter other $R_{\left(i,j\right)}$ with a certain transition probability. However, because other $R_{\left(i,j\right)}$ are less likely to move, from the expected traffic volume point of view, a large amount of content can be pre-cached where the probability of moving is high, thereby lowering backhaul traffic. Further, even if the vehicle moved to an $R_{\left(i,j\right)}$ with a transition probability that is sufficiently low, there is no trouble in providing the maximum amount of contents to the vehicle due to the constraint of the expression (10).

The last two constraints in Equations (12) and (13) calculate the maximum content amount that the vehicle can receive from the $R_{\left(i,j\right)}$ within the communication range of the $R_{\left(i,j\right)}$ based on the current speed of the vehicle. The amount of all the pre-cached contents does not need to exceed the amount available to the vehicle. To solve the problem that more backhaul traffic is consumed when caching contents requested by a vehicle to all RSUs, we propose that if the vehicle pre-caches more content than can be provided, the vehicle will not be able to receive all prepared content, so that the traffic that the $R_{\left(i,j\right)}$ could not provide to the vehicle in the traffic consumed for pre-caching content. Therefore, it is not necessary to pre-cache the contents more than the amount available. Through the given optimization formula, the maximum amount of content can be provided to the vehicle while consuming minimum backhaul traffic without waste. Additionally, the total amount of pre-cached contents should be less than the caching capacity of each RSU. To minimize the total traffic, as the amount of pre-caching content could be the negative amount, we have the constraint that each amount should not be negative.

## 4. Performance Evaluation

### 4.1. Simulation Model and Performance Evaluation Metrics

We compare the MDPC with the existing caching schemes by a simulation for performance evaluation. The simulation is implemented on the network simulator NS-3.2.7 [42]. In our scenario, there is a requester vehicle and four RSUs placed at each intersection, and the network area is 1 km × 1 km in size. The content server is connected by a backhaul network with a bandwidth of 1 Mbps and a delay of 10 ms. Additionally, the RSUs are connected to the backhaul network, using P2P and having a data transmission rate of 50 Mbps. The communication between the RSU and the vehicle uses IEEE 802.11p [39] with a data rate of 24 Mbps. The V2I communication range is 100 m. The vehicle sends an interest packet once it enters the communication range of an RSU. The existing caching schemes to be compared are CacheMax [9,34] and CacheAll. CacheMax means the concept where the highest probability RSU caches contents and CacheAll indicates the assumption that all adjacent RSUs cache contents. The result values indicate the average of 100 times of simulation. In addition, the same simulation environment was implemented as mentioned above to compare MDPC and MDPC_TO. The content server is connected by a backhaul network with a bandwidth of 1 Mbps and a delay of 10 ms. Additionally, the RSUs are connected to the backhaul network, using P2P and having a data transmission rate of 1000 Mbps. The communication between the RSU and the vehicle uses IEEE 802.11p [39] with a data rate of 50 Mbps. The V2I communication range is 200 m.

**Figure 5:**
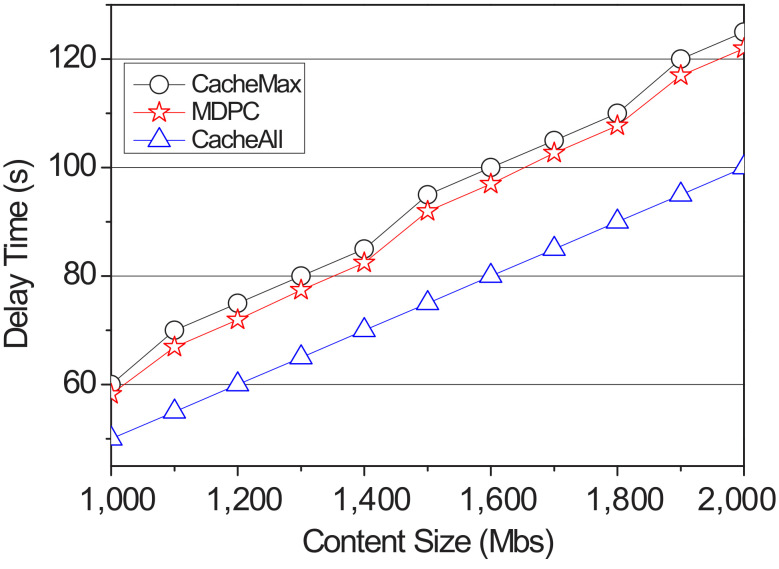
The delay time impacted by content size.

Figure 5 shows the delay time impacted by content size. The main purpose of content caching in content-centric vehicular networks is to reduce the delay time for vehicles to receive content. In our simulation, we evaluate the performance in terms of delay time. The delay time is measured at the time between the time when the content is requested and the time when the last content chunk is received. The delay time does not include the travel time of the vehicle. We fix the hit rate on moving at 50%. The content size requested by the vehicle is increased by 100 Mbs from 1000 Mbs to 2000 Mbs. As expected, in the case of the CacheAll, since the contents are cached in all RSUs on the route, it is possible to receive the last number of the content chunk faster than the CacheMax and the proposed strategy. When CacheMax and the proposed strategy have the same probability of hit rate on moving, the CacheMax has a longer delay time because it has to receive a larger amount of content from the content server instead of the neighbor RSUs.

**Figure 6:**
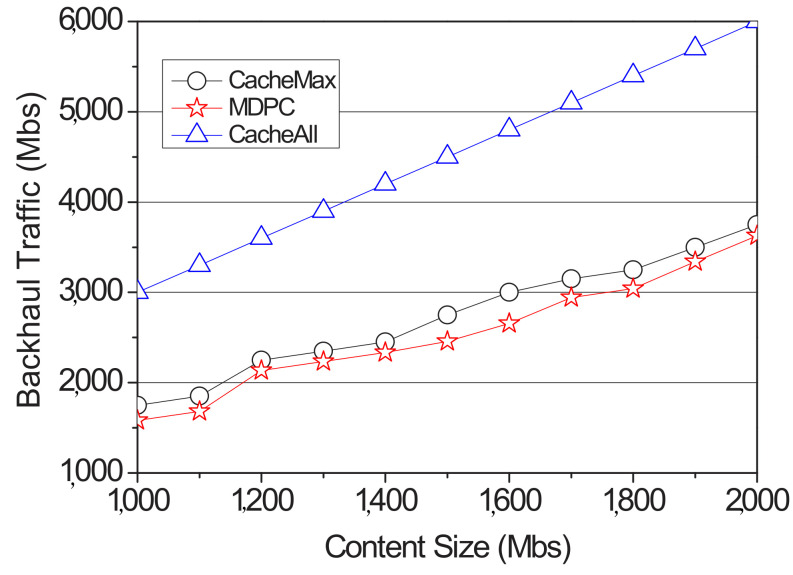
Backhaul traffic of according to the content size.

Figure 6 shows the backhaul traffic on the networks with the proposed strategy and the two schemes. The goal of ICN and proactive caching is to reduce content server traffic. Backhaul traffic considered the traffic generated when transmitting content from the content server to the RSU and the traffic generated when receiving content from the RSU. Although traffic does not occur because the content is not received by the neighbor RSUs in the case of the CacheAll, the traffic volume becomes largest because the content server must generate the traffic for all the contents to all RSUs. In the case of CacheMax, when the content is received by the content server, the same amount of content as the proposal is transmitted. However, when the prediction fails, the traffic volume increases because the entire amount of cached content must be transmitted from the server to the corresponding RSU through the backhaul network. However, in the case of our strategy, the cached content is distributed to all the neighbor RSUs in proportion to the transition probability of movement, so even if a cache miss occurs, the amount of content transmitted through the backhaul becomes smaller than that of the CacheMax.

**Figure 7:**
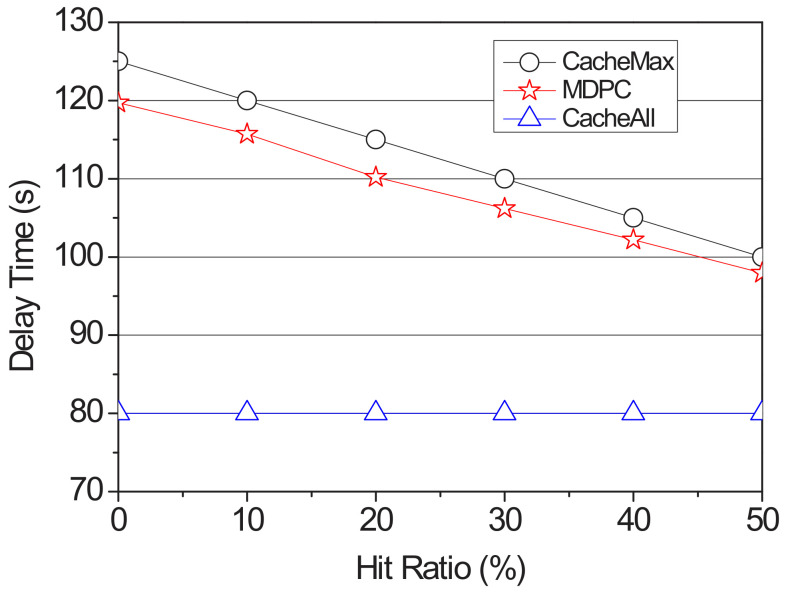
Delay time according to the hit ratio.

We vary the hit ratio from 0% to 50% by 10%. In this simulation, the hit ratio could be defined as the probability to move to the highest RSU. In other words, that means the ratio that the prediction is successful. Figure 7 presents the delay time according to the hit ratio. CacheAll has a constant result because it does not exploit the transition probability and caches content chunks to all RSUs. However, in the comparison between the CacheMax and the proposed strategy, if the prediction in CacheMax fails, it requires additional delay for requesting and receiving the missing contents to the content server. On the other hand, in the proposed strategy, even if the hit rate is low, the content is cached in proportion to the transition probability, so even if a cache miss occurs, the recovery time could be reduced rather than the other schemes as our strategy could get the missing content chunks from the adjacent RSU based on the content distribution information.

**Figure 8:**
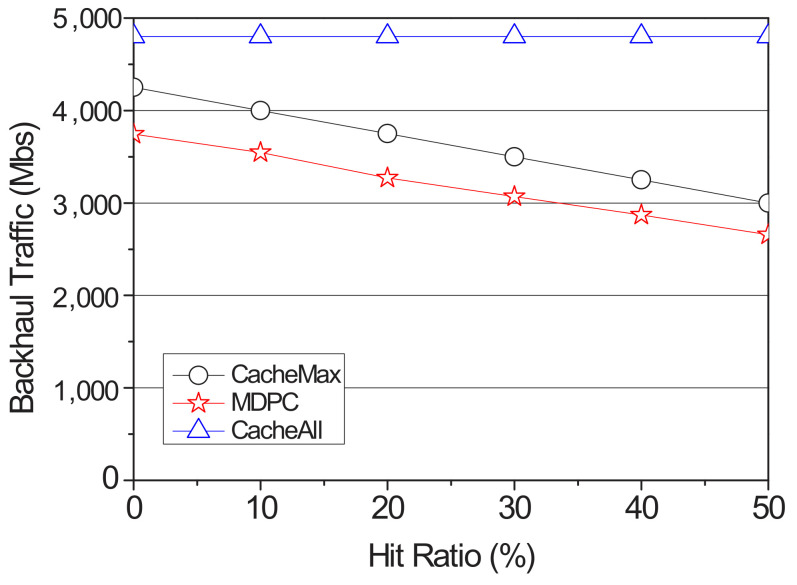
Backhaul traffic according to the hit ratio.

We measure the backhaul traffic by the hit ratio as shown in Figure 8. In the CacheAll, the traffic has uniform values because the contents are cached to all RSUs. Meanwhile, for the recovery of missing content, since the CacheMax requires the participation of many routers in the backhaul networks, it increases the backhaul traffic.

**Figure 9:**
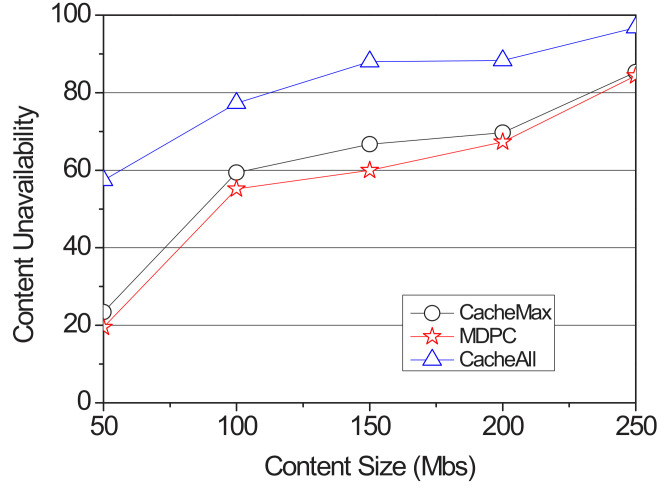
Caching unavailability according to the content size.

Content should be cached efficiently due to the limited storage space of the RSUs. To measure the content availability, we have more request vehicles in the networks. The 63 vehicles send a content request message for storage availability. When a car sends a content request message, the RSU is defined as a hit when caching can be performed compared to the content request size contained in its content storage and content request message. Figure 9 shows the storage failure in accordance with the size of the content. In such a case, content storage quickly dropped when the vehicle sent a content request a message to cache content to all RSUs. Additionally, in the case of CacheMax, although the content is cached to the most probable RSUs when the vehicles move while the car moves when the vehicles gather to the cached RSUs, the storage falls immediately. However, since the proposed strategy distributes and caches contents to the adjacent RSUs, it could support more vehicles and contents.

**Figure 10:**
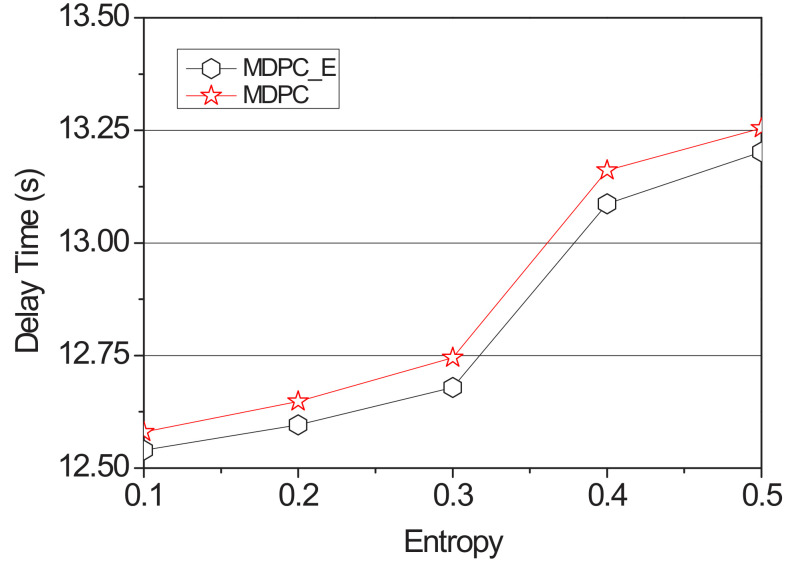
Delay time applying the entropy according to the value of the entropy (0.1–0.5).

**Figure 11:**
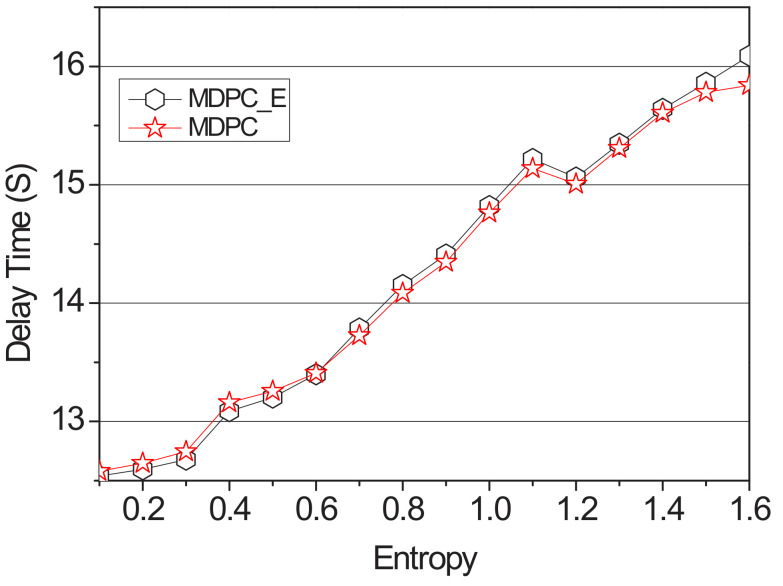
Delay time applying the entropy according to the value of the entropy (0.1–1.6).

Figure 10 and Figure 11 show a graph of the backhaul traffic and delay time of the MDPC and the MDPC_E. It increased entropy from 0.1 to 1.6 by fixing its content size at 2500 Mb, measuring delay time and backhaul traffic.

Figure 10 shows a graph of the delay time measured with the entropy value from 0.1 to 0.5. When the entropy value is less than or equal to 0.5, the possibility of a vehicle visiting the RSU with the highest transition probability is increased. Therefore, MDPC caches content that is cached to the lowest-probability RSU with a weight value to the highest-probability RSU, thus reducing delay time. However, when the entropy value is 0.6 or more as shown in Figure 11, the accuracy is lowered, and the possibility of the vehicle visiting at the highest probability is reduced. Therefore, the delay time will increase as the content cached by the adjacent RSU must be received via the backhaul link.

**Figure 12:**
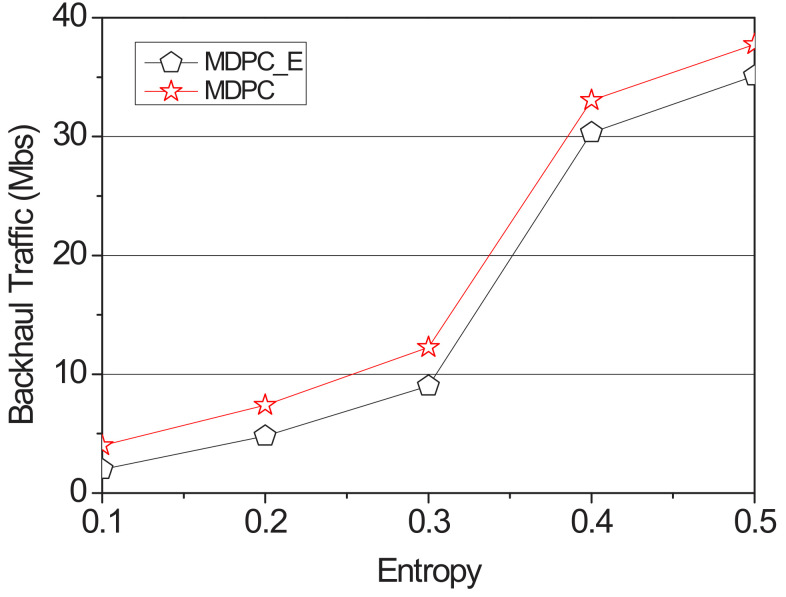
Backhaul traffic applying the entropy according to the value of the entropy.

Figure 12 shows a graph of the delay time measured with the entropy value from 0.1 to 0.5. When the entropy value is 0.5 or less, the accuracy of the probability is 90% or more, so that the possibility of the vehicle visiting the RSU with the lowest probability is reduced. MDPC was not caching content to the lowest-probability RSU but cached the highest-probability RSU with a weight. Therefore, when the vehicle visits the RSU with the highest probability, the RSU reduces the content size received from the adjacent RSU via the backhaul link, thereby reducing the backhaul traffic.

**Figure 13:**
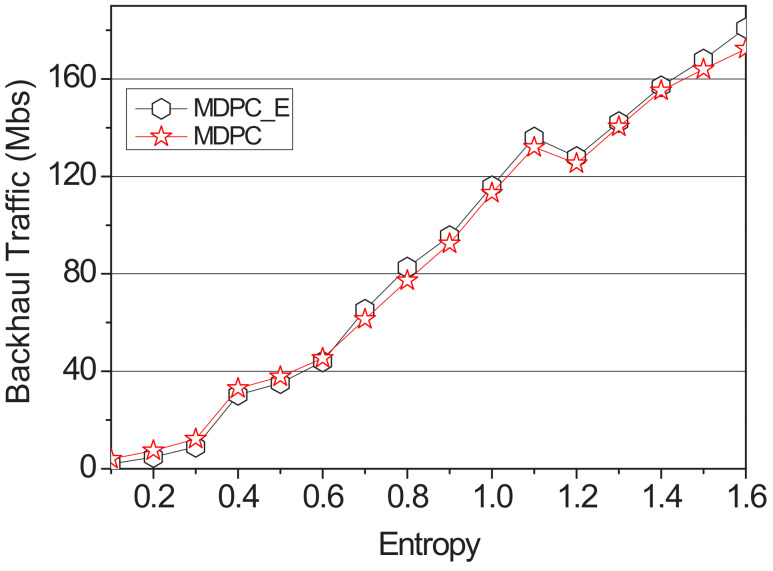
Backhaul traffic applying the entropy according to the entropy.

Figure 13 shows a graph of the delay time measured with the entropy value from 0.1 to 1.6. When the entropy value is 0.6, MDPC and MDPC_E backhaul traffic is almost the same. When the entropy value is 0.6, the backhaul traffic of MDPC and MDPC_E becomes almost the same. However, when the entropy value is 0.6 or more, the accuracy of the probability is reduced, and the possibility of the vehicle visiting the RSU having the highest probability is reduced. Therefore, if the entropy is 0.6 or higher, the backhaul traffic of MDPC is higher than the backhaul traffic of MDPC_E.

### 4.2. Simulation Results of MDPC_TO

In this section, we compare the backhaul traffic and delay time performance of MDPC and MDPC_TO. The MDPC strategy caches content in proportion to the transition probability derived from the Markov probability of the vehicles. As the next step, the MDPC_TO strategy optimizes the transition probability derived from the Markov model from the perspective of backhaul traffic.

**Figure 14:**
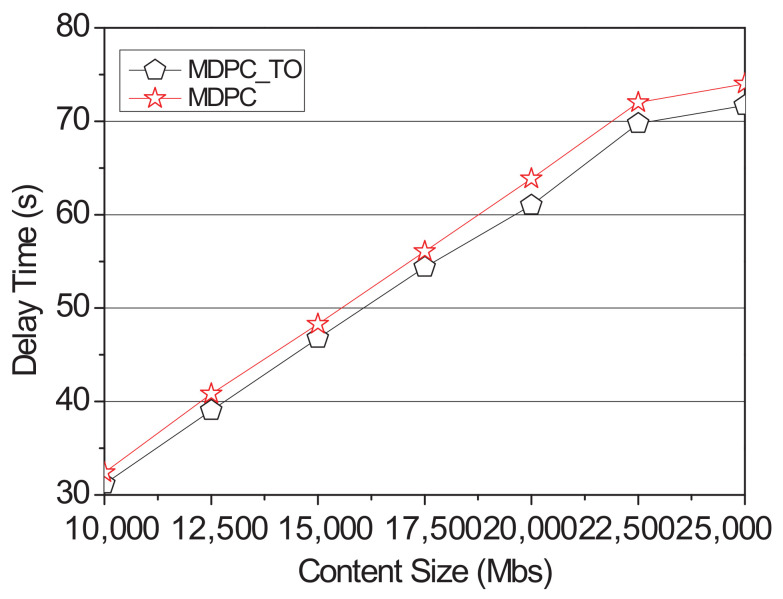
Delay time of optimization according to the content size.

Figure 14 compares the performance differences between the delay time of MDPC and MDPC_TO. Delay time occurs when the cached content is transmitted to the vehicle to which the RSU has sent the request or the content is requested to the adjacent RSU and the content is being transmitted from the adjacent RSU. MDPC_TO has a lower delay time than MDPC because it is optimized where the delay time is minimized for cached contents.

**Figure 15:**
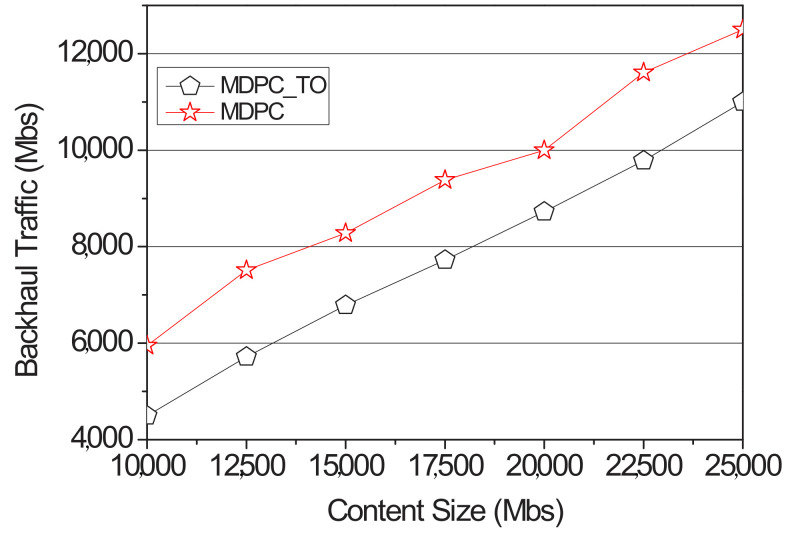
Backhaul traffic of optimization according to the content size.

When the vehicle sends a request to the RSU, the RSU downloads the content on the content server. When requesting content from an RSU adjacent to the RSU, the content is distributed through a backhaul link. Backhaul traffic occurs in this case. Figure 15 shows a graph related to backhaul traffic of MDPC and MDPC_TO. MDPC_TO minimizes the content size requested by the neighboring RSU from the perspective of backhaul traffic, resulting in lower backhaul traffic than MDPC.

**Figure 16:**
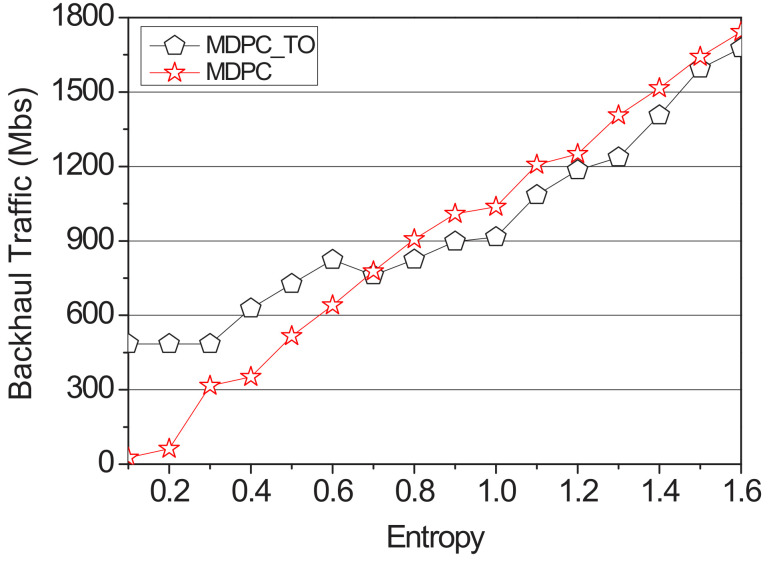
Backhaul traffic of optimization according to the entropy.

Figure 16 shows a comparison of the backhaul traffic between MPDC and MPDC_TO according to the entropy value. The simulation used the random value function of NS-3 to distribute the transition probability. Additionally, the content size is fixed to 2500 Mb, and the simulation is performed. The entropy value was measured using Formula (4) and increased by 0.1 from the minimum value of 0.1 to the maximum value of 1.6 in the range to measure backhaul traffic. MDPC_TO does not have the entry value applied to the objective function, so when the entry value is low, backhaul traffic is higher than MDPC. However, backhaul traffic of MDPC_TO and MDPC gradually becomes similar in 0.7 sections, which are about 80% accurate, and the difference becomes larger until it reaches 1.6. In the case of MDPC_TO, the content size to be cached in RSU is fixed. When the content size to be pre-cached is calculated by an MDPC strategy, the entropy value is about 0.8. Therefore, the difference is increased from the section of 0.8 or more.

## 5. Conclusions

This paper proposes a distributed pre-caching strategy (MDPC) in a content-centric vehicular network for increasing vehicles and contents. To reduce the redundancy of contents on the networks, we exploit the transition probability for the distribution of contents to the next roadside units (RSUs). In order to decrease the delay time, the RSUs share the distribution information for the content; then they are able to request the missing content chunks to the other adjacent RSUs rather than the original content server. Via the simulation, we evaluate the performance in terms of delay time, backhaul traffic, and content availability. Further, as a result of comparing the MDPC and the MDPC when the entropy is 0.5 or less, it is proved that it is more efficient not to cache the content to the RSU having the lowest probability. We optimized MDPC (MDPC_TO) to reduce backhaul traffic. The overall performance was increased, but the optimization constraint did not include transition probability, so backhaul traffic was higher than MDPC when the entropy value was lower. In order to solve these problems, a transition probability will be added when setting a constraint condition.

## Figures and Tables

**Table 1:** Content caching parameters.

Notation	Description
v${}_{r}$	Requesting vehicle
$C_{T}$	The last chunk number of the requested content
$R_{i}$	Current RSU
$C_{i}$	The last chunk number provided from $R_{i}$
D${}_{i}j$	The amount of pre-cached to adjacent RSU *j*
$R_{\left(i,j\right)}$	Next RSU after $R_{i}$
*n*	The number of $R_{\left(i,j\right)}$ (*n* = 1, 2, 3, …)
$p_{ij}$	The probability to move from $R_{i}$ to $R_{\left(i,j\right)}$
$C_{ij}$	The chunk number cached in $R_{\left(i,j\right)}$
h	Entropy Value
w	Maximum V2I data transmission rate
CAPA${}_{j}$	The caching capacity of the RSU *j*

**Table 2:** The amount of the content caching according to the probability.

The Identifier of RSU	The Number of the Chunk	The Probability
** $R_{\left(i,j\right)}$ **	** $C_{pre(i,j)}$ **	** $p_{ij}$ **
$R_{\left(1,1\right)}$	204 (241∼444)	0.85
$R_{\left(1,2\right)}$	24 (445∼467)	0.10
$R_{\left(1,3\right)}$	12 (468∼480)	0.05

**Table 3:** Entropy adjusting.

Entropy	Over 0.5		
$R_{ij}$	$R_{11}$	$R_{12}$	$R_{13}$
$C_{ij}$	204→216	24→24	12→0
w	0.05	0	0

## Data Availability

Not applicable.
